# Investigating the cardioprotective potential of quercetin against tacrolimus-induced cardiotoxicity in Wistar rats: A mechanistic insights

**DOI:** 10.1515/med-2024-1130

**Published:** 2025-02-24

**Authors:** Ankit Verma, Tarique Anwer, Muzaffar Iqbal, Vinod Gahlot, Roshi Khan, Manju Sharma, Mohammad Sohail Akhtar

**Affiliations:** HIMT College of Pharmacy, Dr. A.P.J Abdul Kalam Technical University (AKTU), Knowledge Park 1, Greater Noida, Gautam Budh Nagar, 201310, U.P, India; Department of Pharmaceutical Chemistry, College of Pharmacy, King Saud University, Riyadh, 11451, Saudi Arabia; Department of Pharmacology, School of Pharmaceutical Education & Research, Jamia Hamdard, New Delhi, 110062, India; School of Pharmacy, College of Health Sciences, University of Nizwa, Nizwa, Oman

**Keywords:** cardiotoxicity, cytokines, quercetin, tacrolimus, lipid peroxidation

## Abstract

**Purpose:**

The aim of this research study is to assess the ability of quercetin to protect the heart from the negative consequences of tacrolimus-induced cardiotoxicity.

**Methods:**

A total of 30 rats were divided into 5 groups. Tacrolimus was used to induce cardiotoxicity, whereas quercetin was employed as a protective agent.

**Results:**

Tacrolimus administration significantly raised the levels of serum cardiac biomarkers (Lactate dehydrogenase, creatine kinase-myocardial band, and troponin-I) as well as inflammatory biomarkers (tumor necrosis alpha and interleukin 6). The administration of quercetin reduced raised levels of cardiac and inflammatory biomarkers significantly. In addition, treatment with tacrolimus resulted in higher malondialdehyde (MDA) (lipid peroxidation marker) levels and falling in the levels of reduced glutathione (GSH) as well as antioxidant enzymes such as superoxide dismutase (SOD), glutathione reductase (GR), and catalase (CAT). Quercetin treatment significantly reduced MDA levels and increased GSH and antioxidant enzyme (SOD, GR, and CAT) levels. Moreover, the tacrolimus-administered group exhibited histological changes in cardiac tissue cited as vacuole formation, large and uncondensed nucleus, and cardiomyocyte hypertrophy. The quercetin treatment reduced the inflammatory cell infiltration in cardiac tissue and thus reduced vacuole formation and hypertrophy.

**Conclusions:**

The outcome showed quercetin’s cardioprotective potential against tacrolimus-administered cardiotoxicity. Consequently, it is concluded that quercetin may be used as add-on therapy with tacrolimus to reduce cardiac adverse effects.

## Graphical abstract

**Figure:**
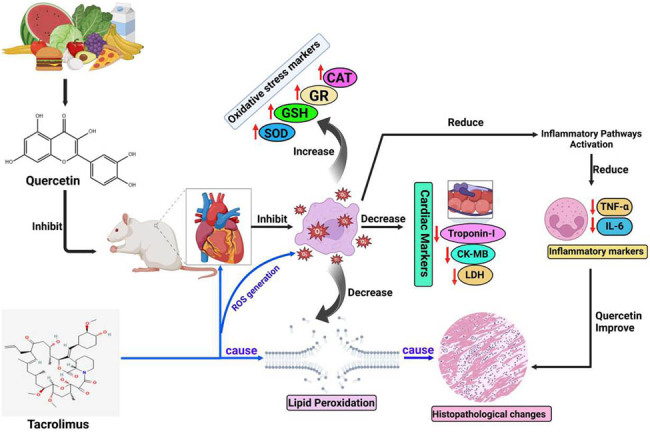

## 1 Introduction

Cardiotoxicity is defined as an abnormality in the heart muscle or its function [1]. It occurs mainly due to inhalation or consumption of certain chemicals or treatment with some drugs [2]. Drug-induced cardiotoxicity is a severe clinical issue that arises when certain drugs are administered and harm the heart [3]. Heart failure, hypertension, myocardial ischemia, arrhythmias, and abnormalities in cardiac tissue structure and integrity are signs of cardiotoxic consequences [4]. The immunosuppressive drug tacrolimus belongs to a class of medication that is used in patients who undertook organ transplantation to avoid rejection. It is also used to treat particular inflammatory disorders, exerting therapeutic effects by modifying the immune system [5]. Being an immunosuppressive medication, it is primarily employed to prevent post-transplantation organ rejection and also to control inflammatory conditions like eczema and psoriasis [6,7]. Tacrolimus effectively dampens the immune response by inhibiting T-cell activation and hence making it a valuable agent in preventing organ rejection following transplantation [8]. Its therapeutic index is very narrow, making it challenging to choose the correct and precise dose, as it influences the concentration of active drugs in the bloodstream [9].

Tacrolimus enhances the production of proinflammatory cytokines and endothelial activation markers in cultured murine endothelial and vascular smooth muscle cells by inducing TLR4 (Toll-like receptor). This activity regulates reactive oxygen species (ROS) generation and nuclear factor kappa B (NF-κB) pathway (namely IκBα and p65 phosphorylation), which further promotes the synthesis of proinflammatory factors [10]. These findings strongly support and confirm the theory that tacrolimus exposure to endothelial cells triggers TLR, which further leads to NF-κB activation and expression of proinflammatory genes [11]. Previous research studies have reported that TLR4 directly activates the master regulatory molecule Akt (serine/threonine-specific protein kinase), leading to its conversion into pAkt, which then further triggers the production of ROS [12,13]. Unfortunately, the exact mechanism behind Tacrolimus-mediated cardiotoxicity is still unknown. Several researchers have proposed different hypotheses regarding its mechanisms, including toxicity related to metabolites, accelerated apoptosis, and intensified inflammation [14].

Quercetin is a natural flavonoid (a polyphenolic compound) that is found in high amounts in a variety of fruits, vegetables, and cereals. It is considered one of the most popular dietary flavanols in the diet of the Western region. It exhibits therapeutic effects by interacting with various molecular targets, particularly by influencing antioxidant, anti-inflammatory, and anticancer pathways. It has incredible antioxidant and anti-inflammatory properties [15,16]. Bioactive compounds from foods like apples, onions, berries, and even green tea could significantly dampen the risk of cardiovascular complications and cancer as well as boost our immune system [17]. Consuming quercetin has been shown to counteract the formation of ROS and the alteration of mitochondrial defects. One of the reasons for gaining interest is because it scavenges free radicals and protects cells from damage [18]. Quercetin acts primarily on leukocytes to inhibit the activation of immune cells, preventing the release of pro-inflammatory cytokines and chemokines that promote swelling. It accomplishes this by interacting with several intracellular signaling molecules, namely kinases, membrane proteins, phosphatases, and enzymes that are important for specific cellular function [19]. Previous studies have reported the protective effect of quercetin in renal damage after cyclophosphamide administration [16]. According to their research, quercetin attenuated cyclophosphamide-induced kidney damage by controlling the expression of apoptotic and inflammatory factors. Quercetin substantially exhibited anticancer effects in both *in vitro* and *in vivo* settings by augmenting apoptosis of CT-26, LNCaP, MOLT-4, and Raji cell lines [20]. A previous study revealed that quercetin showed a protective effect against liver damage caused by paracetamol [21]. Another researcher demonstrated quercetin’s anti-inflammatory properties in an experimental model of rheumatoid arthritis [22]. In a research study, Lokman et al. have documented the preventive benefits of quercetin against 5-FU-induced cardiotoxicity [23]. Kumar et al. have demonstrated that quercetin protects cardiac tissue from damage in isoproterenol-induced myocardial infarction in Wistar rats [24]. Quercetin also plays a major role in preventing osmotic stress-induced cytotoxicity in *in-vitro* cardiomyocyte H9c2 cell line model by modulating cytosolic calcium level and mitochondrial membrane potential [25].

Therefore, this study aimed to investigate the protective effects of quercetin on cardiac tissue against tacrolimus-treated cardiotoxicity in experimental animals. The study also aimed to explore the connection between quercetin, oxidative stress, and pro-inflammatory mediators in the heart after tacrolimus treatment.

## 2 Materials and methods

### 2.1 Drugs and chemicals

**Figure 1:**
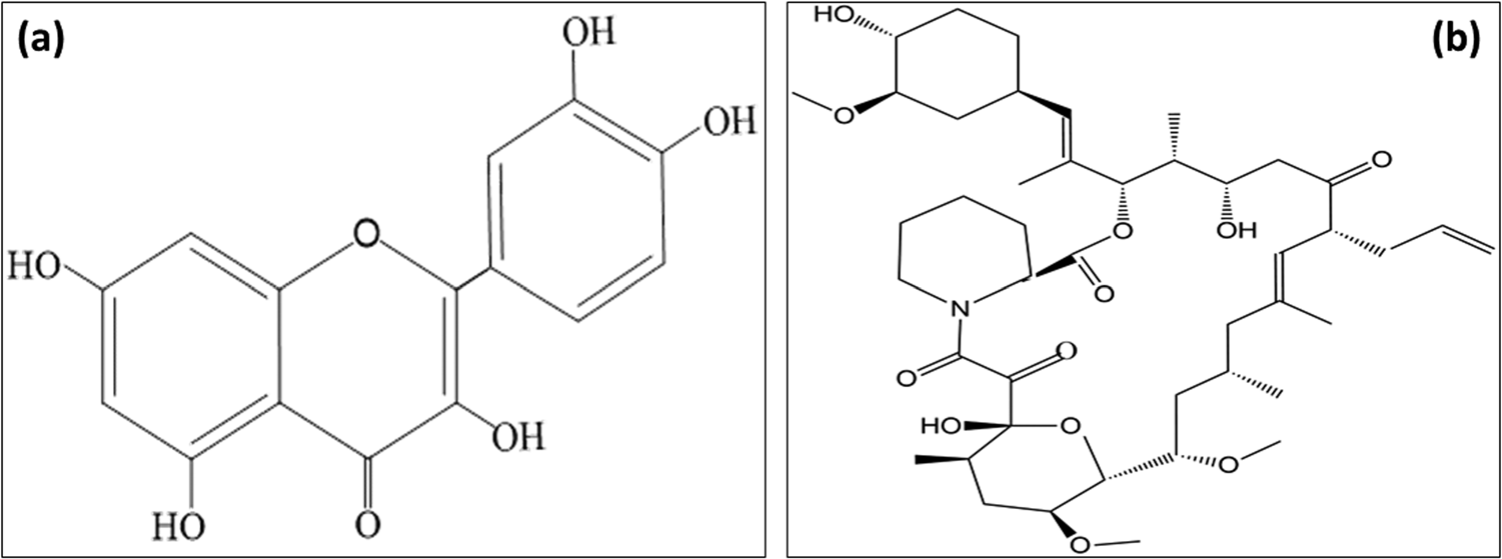
The structure of quercetin (a) and tacrolimus (b).

Quercetin was acquired from the Oxford Lab Fine Chem Ltd (Maharashtra), whereas tacrolimus was a gift sample from Alkem Laboratories (Mumbai). Lactate dehydrogenase (LDH) and creatine kinase-myocardial band (CK-MB) kits were purchased from Accurex Biomedical, Mumbai. The structure of quercetin and tacrolimus is displayed in Figure 1. Tumor necrosis alpha (TNF-α) and interleukin 6 (IL-6) assay kits were obtained from Krishgen Biosystem. Many important chemicals such as TCA, TBA, DTNB, Folin phenol reagent, NBT, NADPH, and GSSG were purchased from Sisco Research Laboratories Pvt, Ltd (Mumbai). All other chemicals used in the research study were purchased from an ISO-certified chemical company.

### 2.2 Experimental animals

IAEC of the College and CCSEA have approved our research protocol (Protocol no. 2024/HIMT/IAEC/FB/001). After approval, we purchased 30 healthy male Wistar rats (150–200 g) from Rodent Research Pvt, Ltd (Jind, Haryana). All animals were kept in quarantine for 7 days at a constant temperature, maintained under a 12 h light/dark cycle, and provided free access to water and food *ad libitum*.

### 2.3 Experimental design

**Figure 2:**
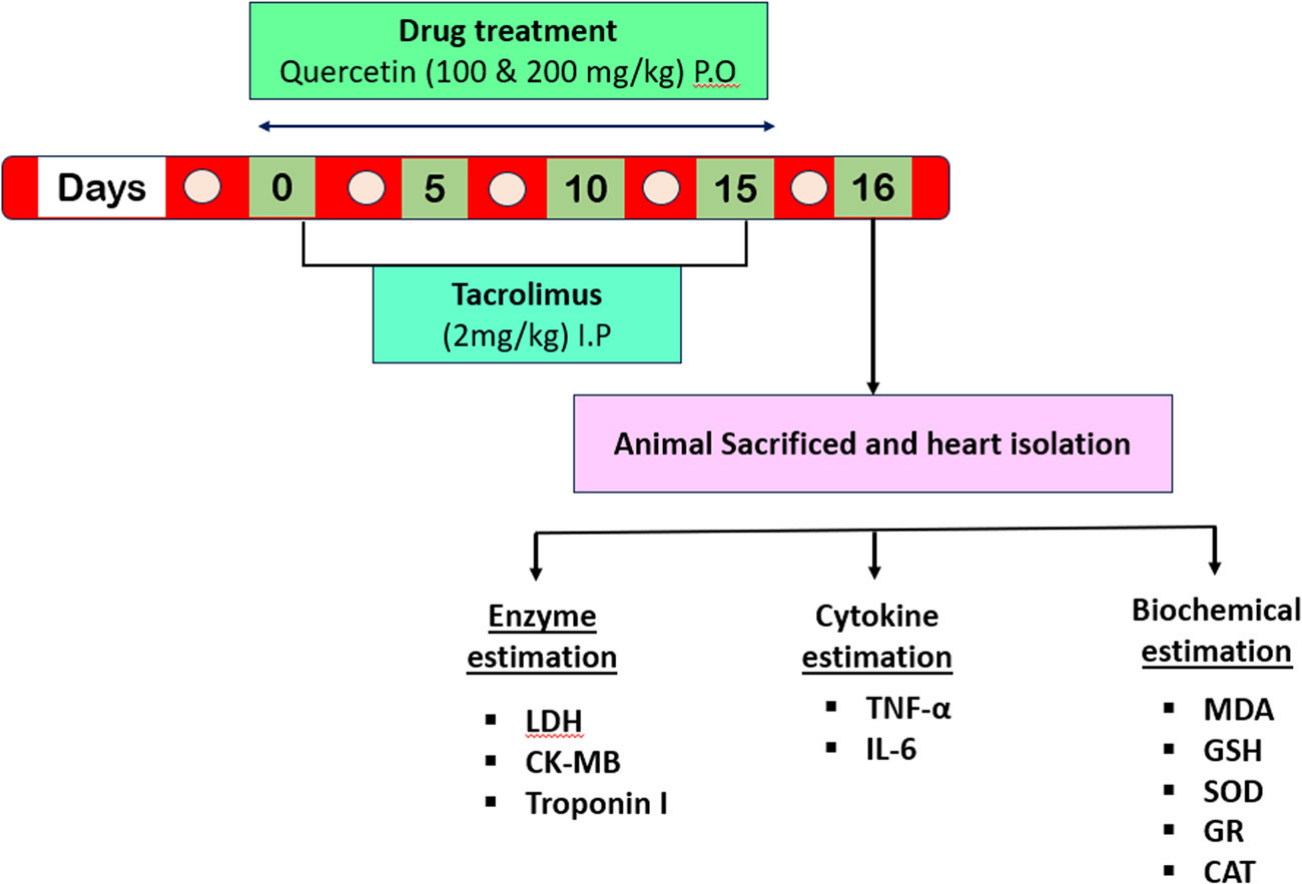
The experimental protocol design.

The experimental design consisted of five groups comprising six animals in each group (*n* = 6), displayed in Figure 2. Group 1 (saline control): all animals received normal saline (1 ml/kg) for 15 days through the oral route (p.o.). Group 2 (disease control): all animals received tacrolimus (2 mg/kg) through i.p. route for 15 days [26]. Group 3 (drug treatment 1): all animals received tacrolimus (2 mg/kg, i.p.) plus quercetin (100 mg/kg, p.o.) for 15 days. Group 4 (drug treatment 2): all animals received tacrolimus (2 mg/kg, i.p.) plus quercetin (200 mg/kg, p.o.) for 15 days. Group 5 (quercetin only): all animals received only quercetin (200 mg/kg, p.o.) for 15 days. The suspension of quercetin was prepared by adding quercetin in 1% carboxymethylcellulose aqueous solution. Every animal in each group had its weight recorded on days 1, 7, and 15 of the study. Rats were euthanized using thiopental (40 mg/kg, i.p.) followed by exsanguination on the 16th day to obtain blood samples for cytokines analysis and biochemical estimations. The blood samples were centrifuged at 3,000 rpm for 12 min to prepare serum. All samples were analyzed to estimate cardiac biomarkers, including CK-MB, troponin-I, and LDH, as well as markers of inflammatory cytokines such as IL-6 and TNF-α. The heart from each animal was excised, washed in ice-cold normal saline, and homogenized for the evaluation of biochemical oxidative stress markers. The resulting homogenate preparation was further used to evaluate oxidative stress markers, like malondialdehyde (MDA), reduced glutathione (GSH), superoxide dismutase (SOD), glutathione reductase (GR), and catalase (CAT). We have kept part of three heart samples from each group in formalin (10%) for the preparation of histological slides.

### 2.4 Assessment of serum cardiac and inflammatory biomarkers

LDH, CK-MB, and troponin-I estimation in serum was performed using assay kits following the manufacturer’s instructions. The inflammatory biomarker (IL-6 and TNF-α) assessment was carried out in serum samples through a simple ELISA kit method according to manufacturer instructions. A 96-well microplate pre-coated with specific antibodies of TNF-α and IL-6 was used and the dilution of standards of both cytokines was prepared according to the instruction of the manufacturer. Then, test samples were carefully added to each well of the pre-coated plate microplate. Subsequently, each microplate well was filled with the horseradish peroxidase (HRP) conjugates and biotinylated detection antibody, which were then incubated at 37°C. After the incubation, the unwanted free component was removed by washing. Then, TMB substrate was added into the well which formed yellowish color, which was measured by an ELISA reader at 450 nm wavelength. The standard curve was used to determine the cytokine concentrations in test samples.

### 2.5 Tissue homogenate preparation

The dissection of the heart was performed using a sterilized dissection kit for homogenate preparation. Phosphate buffer (pH 7.4) and protease inhibitor (1 µg/ml) were used for homogenate preparation. The formed homogenate solution was centrifuged at a temperature of 4°C for 5 min at 800 × *g*. The formed supernatant was separated into another tube and used for LPO and GSH estimation. To isolate the post-mitochondrial supernatant (PMS), the residual homogenate was centrifuged at 10,500 × g for 15 min under a maintained temperature of 4°C. The formed PMS was used for SOD, GR, and CAT assays.

### 2.6 Assessment of cardiac oxidative stress markers

The method described by Ohkawa et al. was used for the estimation of lipid peroxidation [27]. MDA is the end product of LPO and is a significant oxidative stress marker. The result was expressed as *n* moles of TBARS formed equivalents per mg of protein. Glutathione is a vital antioxidant that neutralizes the free radicals generated inside the body and protects them from oxidative stress damage. Its level in the heart was measured through the procedure proposed by Jollow et al. [28]. The outcomes were represented as µ moles of GSH per mg of protein. SOD is an essential antioxidant enzyme that functions to breakdown of superoxide radicals (O_2_˙^−^) into oxygen (O_2_) and hydrogen peroxide (H_2_O_2_). This process protects cells from oxidative damage. Kono et al. method was used to estimate the level of SOD in heart tissue [29]. GR is an essential component of the antioxidant defense mechanism, it is responsible for preserving the equilibrium of the redox state inside the cell. It acts as the catalyst that helps in the conversion of glutathione disulfide (GSSG) (oxidized form) to glutathione sulfhydryl form (GSH) (reduced form), which is necessary for detoxifying reactive oxygen species and preventing oxidative damage to cellular components. GR activity was determined by using the procedure described by Mohandas et al. [30]. It is an antioxidant enzyme in almost all living organisms. It prevents oxidative damage to cells and stimulates the conversion of H_2_O_2_ into oxygen and water. Luck’s method was used to assay CAT activity [31]. The results were represented as µmoles of H_2_O_2_ decomposed/min/mg of protein.

### 2.7 Histopathological assessment

The heart was removed just after the sacrifice of animals, washed with normal saline, and kept in 10% formalin solution to preserve it from breakdown. The heart was then sliced into pieces of proper and smaller size that were immersed in liquid paraffin and allowed it to form blocks after solidification. Using a microtome, the formed blocks were subsequently utilized to create 3–5 µm thick sections. Hematoxylin and eosin stain was used to stain and prepare the slides of these slices for further histological examination at 40× magnification under a microscope.

### 2.8 Statistical analysis

Graph Pad Prism Version 10.2 program was used to analyze the result of the study. Each test sample was conducted in triplicate and their mean values were taken. To assess statistical significance, one-way analysis of variance was applied to the mean data, and then, Tukey’s post hoc test was performed. The analyzed results were compared and shown as standard error mean. A minimal criterion of *p* < 0.05 was established for the findings to meet statistical significance.

## 3 Results

### 3.1 Quercetin and serum cardiac biomarkers

The level of serum cardiac biomarkers like LDH, CK-MB, and troponin-I was found to be significantly elevated (*p* < 0.001) in group 2 compared to group 1 as shown in Table 1. The administration of quercetin (100 and 200 mg/kg) in groups 3 and 4 demonstrated a significant decrease in the levels of LDH, CK-MB, and troponin-I compared to group 2 (*p* < 0.001, *p* < 0.0001). Quercetin alone in higher dose (group 5) demonstrated no significant changes in the levels of cardiac biomarkers compared to group 1, indicating no drug-related toxicity (*p* > 0.05).

**Table 1:** Represent the level of cardiac function parameters

Groups	Treatment	LDH	CK-MB	Troponin-I
1.	Normal control	49.33 ± 2.43	118.02 ± 6.63	1.19 ± 0.20
2.	Disease control (tacrolimus 2 mg/kg, i.p.)	106.85 ± 1.70^###^	439.95 ± 3.51^###^	4.74 ± 0.16^####^
3.	Treatment 1 (tacrolimus 2 mg/kg, i.p. + quercetin 100 mg/kg, p.o.)	78.86 ± 3.76***	346.70 ± 4.46**	3.26 ± 0.67***
4.	Treatment 2 (tacrolimus 2 mg/kg, i.p. + quercetin 200 mg/kg, p.o.)	75.38 ± 3.24***	273.82 ± 5.18**	1.32 ± 0.10****
5.	Only quercetin (200 mg/kg, p.o.)	54.94 ± 5.37	154.32 ± 3.10	1.13 ± 0.98

Group 2 (disease control) equated with group 1 (normal control) shows ^###^ *p* < 0.001 and ^####^ *p* < 0.0001. Groups 3 and 4 (drug treatments 1 and 2) equated with group 2 (disease control) show ***p < 0.001, **p < 0.01, and ****p < 0.0001.

### 3.2 Quercetin and inflammatory biomarkers (TNF-α and IL-6) level

**Figure 3:**
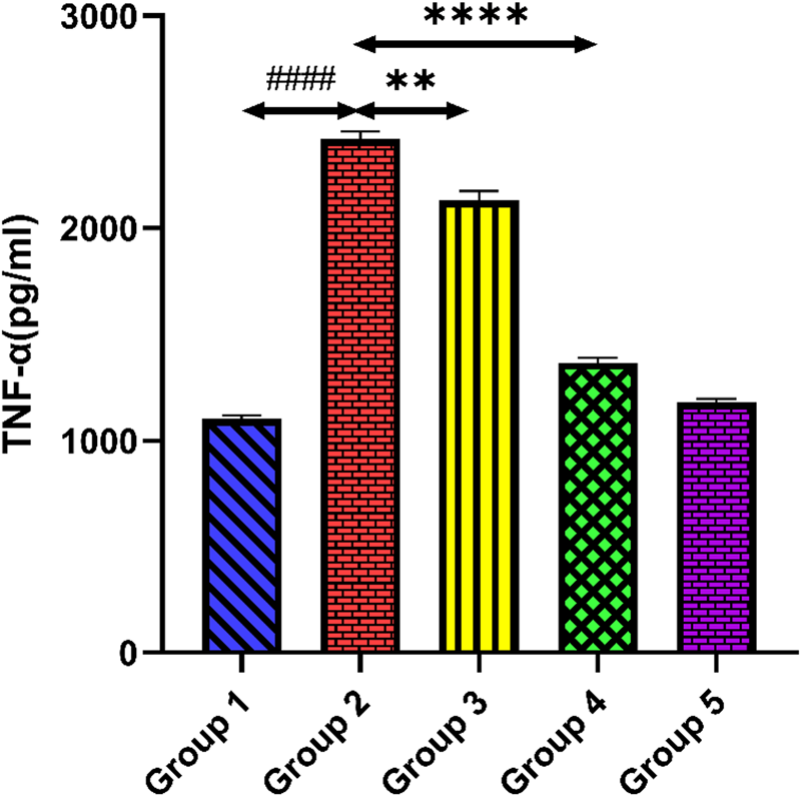
Outcomes of quercetin administration on TNF-α level against tacrolimus-induced cardiotoxicity. Group 2 (diseased control) compared with group 1 (normal control) shows ####*p* < 0.0001. Groups 3 and 4 (treatment control) equated with group 2 (diseased control) show **p < 0.01 and ****p < 0.0001.

**Figure 4:**
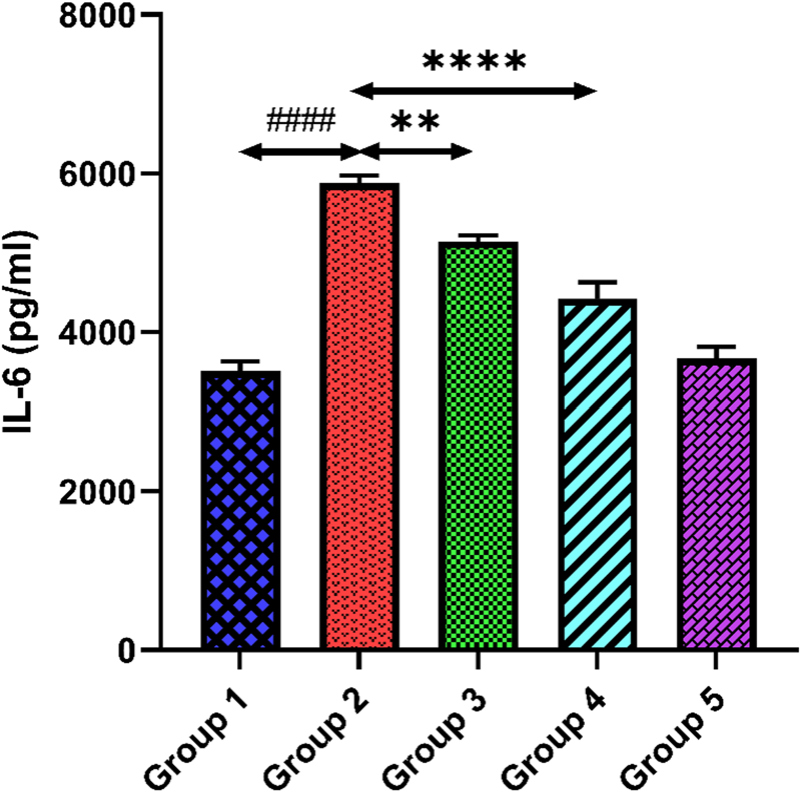
Outcomes of quercetin administration on IL-6 level against tacrolimus-induced cardiotoxicity. Group 2 (diseased control) compared with group 1 (normal control) shows ^####^ *p* < 0.0001. Groups 3 and 4 (treatment control) equated with group 2 (diseased control) show **p < 0.01 and ****p < 0.0001.

Figures 3 and 4 demonstrate TNF-α and IL-6 levels of all groups. Tacrolimus-treated group 2 showed higher levels of TNF-α and IL-6 matched with group 1 (*p* < 0.0001). When quercetin (100 and 200 mg/kg) was administered to groups 3 and 4, it demonstrated a significant reduction in TNF-α and IL-6 levels compared to group 2 (*p* < 0.01) (*p* < 0.0001). The higher dose of quercetin in group 5 demonstrated no significant changes in the levels of inflammatory biomarkers compared to group 1 (*p* > 0.05).

### 3.3 Quercetin and MDA level

**Figure 5:**
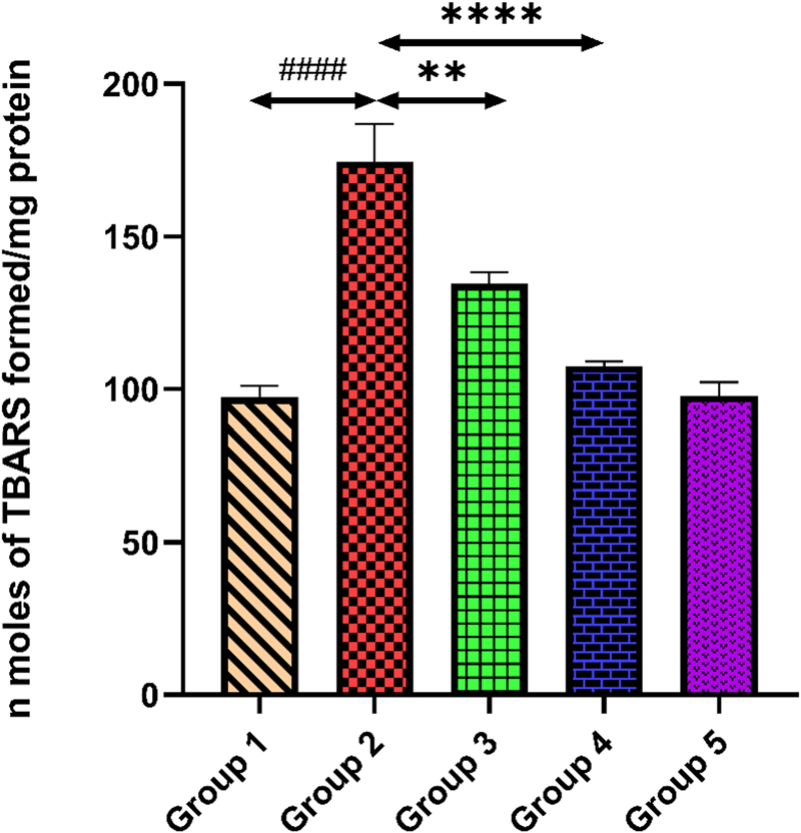
Outcomes of quercetin administration on MDA level against tacrolimus-induced cardiotoxicity. Group 2 (diseased control) compared with group 1 (normal control) shows ####*p* < 0.0001. Groups 3 and 4 (treatment control) equated with group 2 (diseased control) show **p < 0.01 and ****p < 0.0001.

Tacrolimus-treated group 2 showed a higher MDA level in comparison to group 1 (*p* < 0.0001). Quercetin administration in groups 3 and 4 significantly depleted the MDA level equated to group 2 (*p* < 0.001) (*p* < 0.0001). Only quercetin-treated group 5 revealed non-significant changes in the level of MDA compared with group 1, suggesting no drug-related toxicity (*p* > 0.05) and illustrated in Figure 5.

### 3.4 Quercetin and GSH level

**Figure 6:**
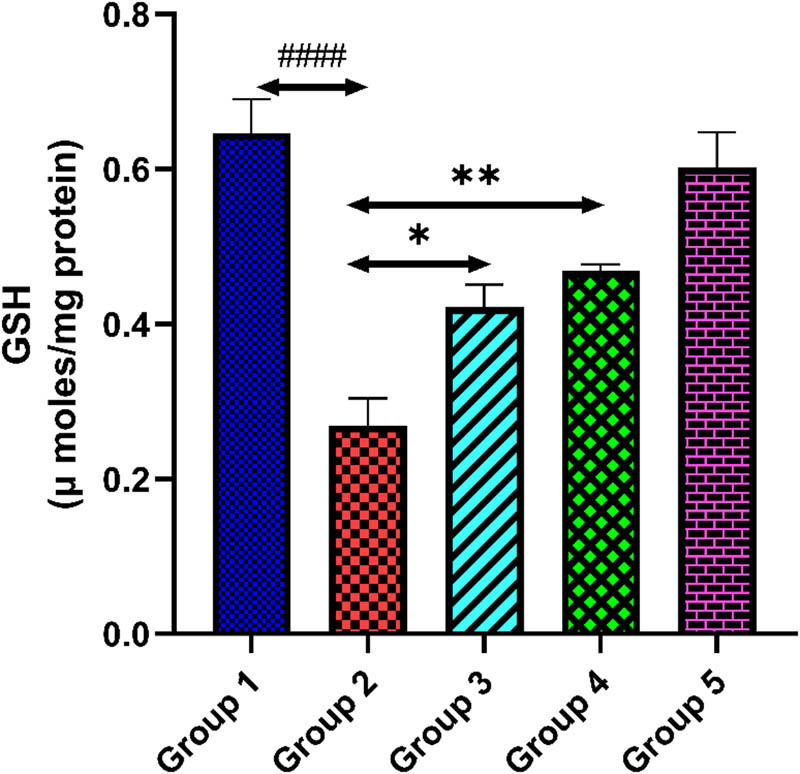
The outcomes of quercetin administration on GSH level against tacrolimus-induced cardiotoxicity. Group 2 (diseased control), when compared with group 1 (normal control), shows a reduced level of GSH, ^####^ *p* < 0.0001. Groups 3 and 4 (treatment control), when compared with group 2 (diseased control), show a significant elevation in GSH level, *p < 0.05 and **p < 0.01.

Figure 6 describes the level of GSH in heart tissue homogenate of all groups. The GSH level in group 2 was reduced when equated with group 1 (*p* < 0.0001). Group 3 and 4 rats when treated with quercetin at doses of 100 and 200 mg/kg showed significantly elevated levels of GSH (*p* < 0.01). However, only quercetin treatment in group 5 exhibited no significant changes in the level of GSH, indicating no drug-related toxicity (*p* > 0.05).

### 3.5 Quercetin and activities of antioxidant enzyme

**Figure 7:**
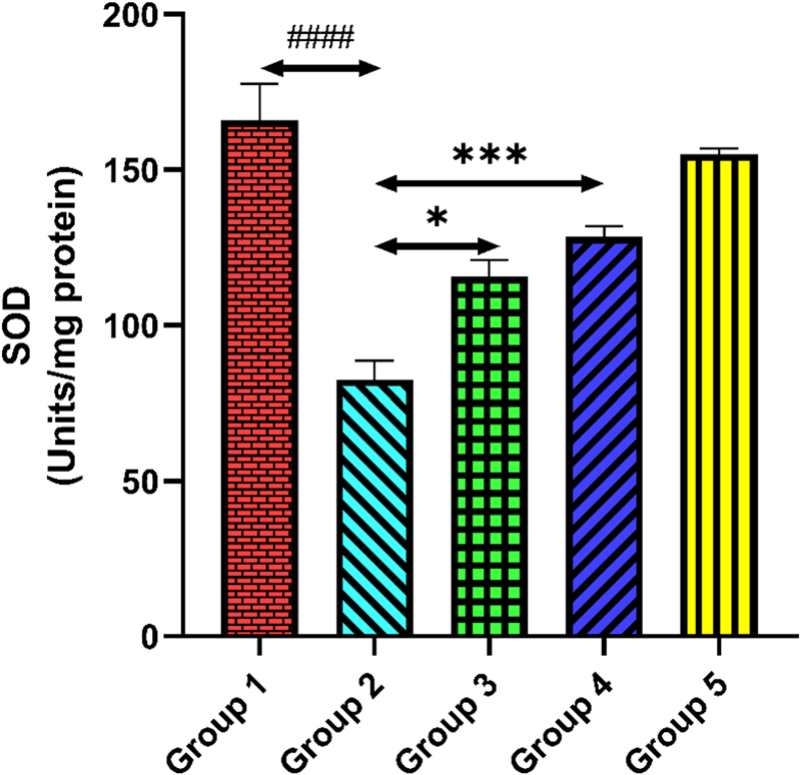
Group 2 (diseased control), when compared with group 1 (normal control), shows a reduced level of SOD, ^####^ *p* < 0.0001. Groups 3 and 4 (treatment control), when compared with group 2 (diseased control), show a significant elevation in SOD level, *p < 0.05 and ***p < 0.001.

**Figure 8:**
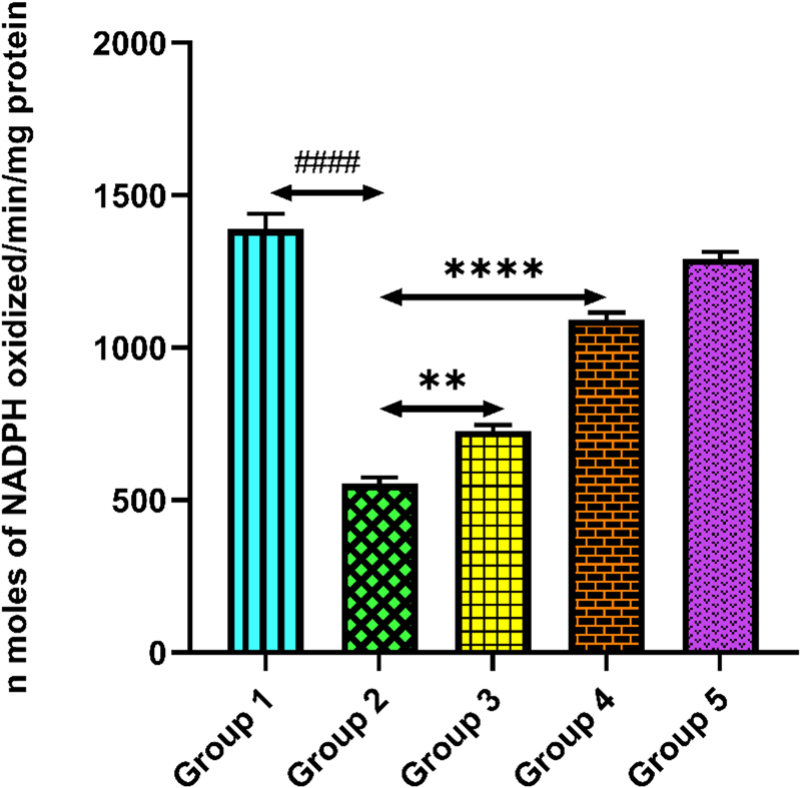
Group 2 (diseased control), when compared with group 1 (normal control), shows a reduced level of GR, ^####^ *p* < 0.0001. Groups 3 and 4 (treatment control), when compared with group 2 (diseased control), show a significant elevation in GR level, **p < 0.01 and ****p < 0.0001.

**Figure 9:**
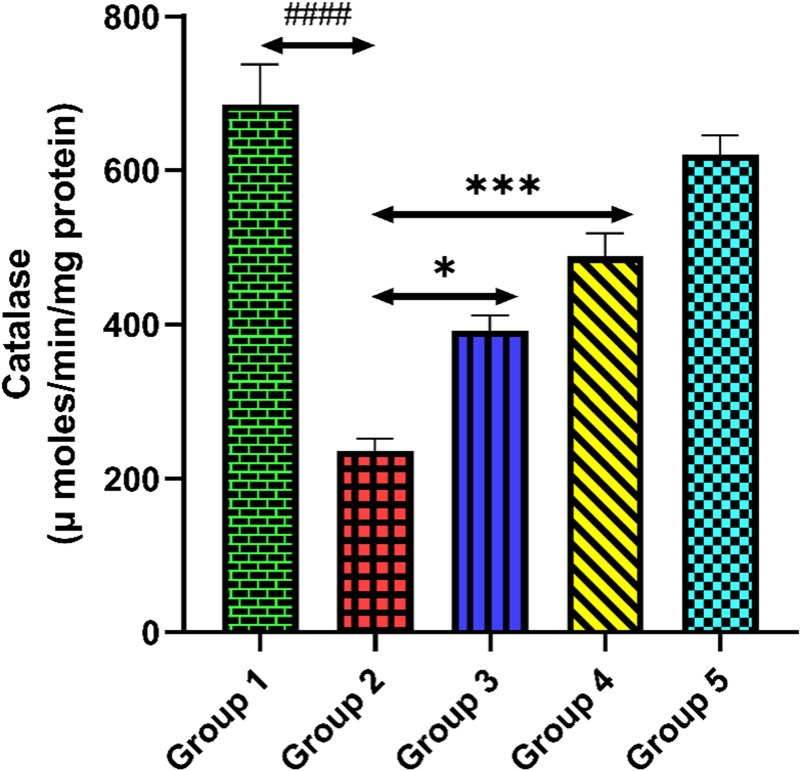
Group 2 (diseased control), when compared with group 1 (normal control), shows a reduced level of CAT, ^####^ *p* < 0.0001. Groups 3 and 4 (treatment control), when compared with group 2 (diseased control), show a significant elevation in CAT level, *p < 0.05 and ***p < 0.001.

The activity of antioxidant enzymes (SOD, GR, and CAT) in the cardiac tissue homogenate is shown in Figures 7–9. The activities of antioxidant enzymes were significantly reduced in tacrolimus-treated group 2 compared to group 1 (*p* < 0.0001). However, quercetin administrations significantly enhanced the activities of antioxidant enzymes compared to group 2 (*p* < 0.001). Only quercetin-treated group 5 rats exhibited no significant changes in the activities of antioxidant enzymes equated to group 1, demonstrating no drug-related toxicity (*p* > 0.05).

### 3.6 Quercetin and histopathological assessment

**Figure 10:**
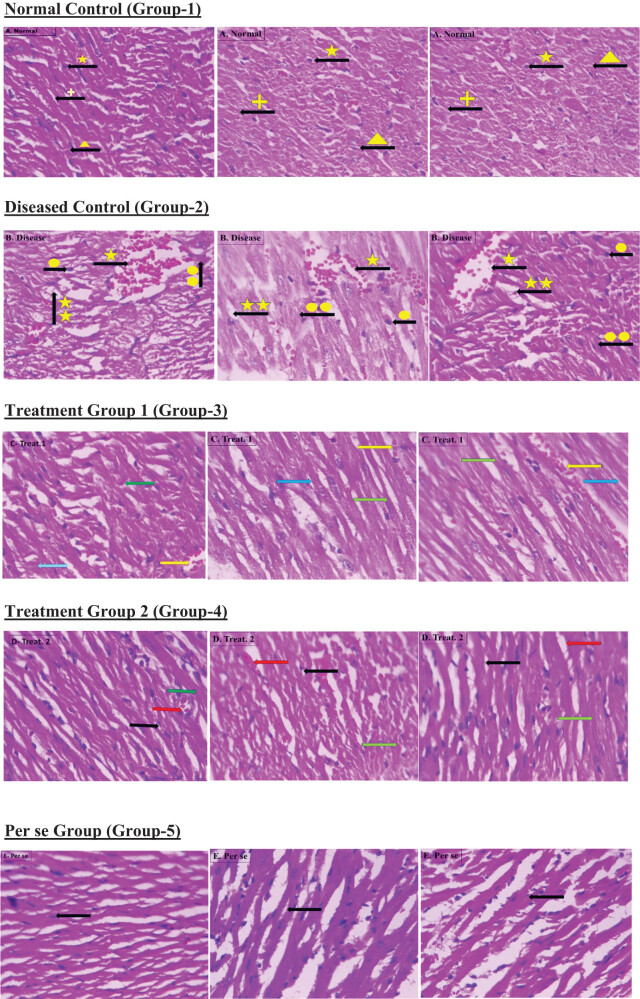
Heart histology of normal control, diseased control, treatment control, and pierce groups. Section of heart tissue: (a) normal control, showing normal cardiomyocyte (yellow star), fibroblast cell (positive yellow sign), and intercalated disc (yellow triangle). (b) Tacrolimus-treated control, showing inflammatory cell (yellow single star), vacuole formation (double yellow star), large and uncondensed nucleus (yellow single circle), and cardiomyocyte hypertrophy (double yellow circle). (c) Quercetin (100 mg/kg) + tacrolimus (2 mg/kg) treated showing the presence of few inflammatory cells (yellow arrow), small vacuole (green arrow), and cardiac hypertrophy (cyan arrow). (d) Quercetin (200 mg/kg) + tacrolimus (2 mg/kg) treated showing reduced hypertrophy of cardiomyocyte (green arrow) and no inflammatory cells present (red arrow) and there are no vacuoles (black arrow). (e) Only quercetin treated (200 mg/kg) showing normal cardiomyocyte with fibroblast cell.

Histopathological examination of group 1 and group 5 manifested a normal structure of cardiac tissue-like normal cardiomyocytes without inflammation. Tacrolimus-treated group 2 signifies a degenerative change in the cardiomyocyte, infiltration of inflammatory cells with vacuolization, and condensed nucleus. Administration of quercetin (100 and 200 mg/kg) in groups 3 and 4 restored the degenerative changes in the cardiac tissue demonstrated in Figure 10.

## 4 Discussion

Tacrolimus is used clinically for the management of immune response against organ transplantation. In other words, we can say that tacrolimus is an immunosuppressive agent used in organ transplantation management. Tacrolimus is reported to have several adverse effects when used clinically. Cardiotoxicity is one of the reported adverse effects caused by tacrolimus. The dosage of tacrolimus administered in this study to produce cardiac toxicity has been determined using prior research investigations done by Hosseini et al. [32]. They have reported that a 2 mg/kg dose of tacrolimus was administered through the intra-peritoneal route for 14 days to cause cardiotoxicity in rats. Oxidative stress, inflammation, and necrosis are the essential mechanisms that may harm cardiomyocytes and cause necrosis or apoptosis [33]. Tacrolimus might also disturb cardiac cell calcium homeostasis mechanism, which can impede regular contraction and relaxation cycles and possibly result in arrhythmias [34]. Additionally, tacrolimus changes endothelial function by raising the synthesis of endothelin-1 and decreasing the synthesis of nitric oxide (NO), which exacerbates hypertension and vascular dysfunction [35]. Moreover, tacrolimus suppresses the mTOR pathway, which is essential for cell proliferation and survival and may cause cardiomyocyte hypertrophy and fibrosis [36]. It has been speculated by researchers that intermediates of tacrolimus formed after hepatic metabolism via the CYP3A enzyme affect the cardiac tissue [13,37]. The exact mechanism through which tacrolimus can cause cardiotoxicity is still unknown.

In cardiotoxicity, the level of cardiac biomarkers like LDH and CK-MB is elevated due to myocardial tissue damage and cellular destruction. To determine the degree and severity of cardiotoxicity, high LDH and CK-MB levels are often assessed with other markers for clinical evaluations [38]. Cardiotoxicity results in cardiac cell damage via mechanisms including oxidative stress, inflammation, and disruption of cellular processes, regardless of whether medications like tacrolimus or other reasons induce it. Damage of this kind may lead to necrosis, apoptosis, or disintegration of the cell membrane, releasing intracellular substances into the circulation, including troponin-I. Thus, elevated troponin-I levels are an essential marker for diagnosing and tracking cardiotoxicity because they are a sensitive and specific sign of heart damage.

A previous research study reported the cardioprotective potential of Olmesartan and Aliskiren in tacrolimus-administered cardiotoxicity in rats [26]. Hosseini et al. investigated the cardioprotective potential of pomegranate seed oil against tacrolimus-administered cardiotoxicity [32]. In this investigation, we also found a significant elevation in the LDH, CK-MB, and troponin-I levels in serum after tacrolimus treatment, indicating a sign of cardiotoxicity. Quercetin administration significantly reduced the LDH, CK-MB, and troponin levels. A previous study reported that quercetin showed cardioprotection against doxorubicin cytotoxicity by stimulating the repair mechanism of cardiomyocyte damage [39].

Oxidative stress is one of the main factors that has been implicated in the pathogenesis of tacrolimus-induced cardiac toxicity [40]. Tacrolimus after metabolism generates multiple metabolites which accumulate in the cardiac cells and initiate ROS generation [41]. The tacrolimus induced generated ROS damages structures and function of cardiac tissue mainly through lipid peroxidation and deduction of the antioxidant enzyme system. MDA is the final product of lipid peroxidation. Several studies on oxidative stress damage reported that LPO is the major marker that can define the oxidative stress condition of any tissue, which ultimately results in the damage of the lipid membrane of cells through the generation of ROS [42]. GSH, also known as GSH, is a powerful antioxidant in almost all human cells. It is essential for maintaining the redox equilibrium and shielding cells from oxidative damage. Its main role is to counteract ROS and free radicals, which may harm cells. GSH is also necessary for immune system support, liver detoxification of toxic chemicals, and the regeneration of other antioxidants, including vitamins C and E [43,44]. The ROS generated after the introduction of tacrolimus reduces the GSH level in the cell, ultimately resulting in an imbalance between the oxidant and antioxidant mechanisms of the cell and thus LPO occurs. In our study, we find the reduced level of GSH in the heart tissue and increased level of MDA that indicate cell death. Quercetin administration on both doses significantly reduced the level of MDA and increased the GSH level, which documented the cardioprotective potential of quercetin against tacrolimus-induced oxidative damage.

One of the most essential enzymes in the body’s defense against oxidative stress is SOD. It converts superoxide radicals into hydrogen peroxide or regular molecular oxygen which is further broken down by glutathione peroxidase and CAT, among other antioxidant enzymes. SOD aids in preventing oxidative damage to cellular components, including DNA, proteins, and lipids, by reducing the damaging effects of superoxide radicals [45]. Alteration in the SOD and CAT levels in cardiac tissue after tacrolimus administration causes an imbalance in the level of superoxide radical and hydrogen peroxide or regular molecular oxygen that ultimately leads to cellular damage through ROS. In a previous research, Majhi et al. reported that quercetin alone and in combination with candesartan ameliorated doxorubicin-induced cardiotoxicity [46]. The administration of quercetin exhibited remarkable elevation in the SOD and CAT levels that showed cardioprotective potential against tacrolimus-induced cardiotoxicity.

GR is one of the important enzymes that is extremely important to the body’s antioxidant defense system. It works by catalyzing the reduction of oxidized glutathione (GSSG) back to GSH and preserving the equilibrium between reduced and oxidized glutathione. Its function provides a continuous supply of GSH, the main antioxidant enzyme that prevents cell damage from free radicals and ROS [47]. Reduction in the level of GR after tacrolimus administration reduces the conversion of oxidized glutathione to GSH. Treatment with quercetin significantly increased the level of GR and thus maintains homeostasis between reduced and oxidized glutathione.

In addition to cellular oxidative stress, activations of inflammatory pathways play a big role in the pathophysiology of tacrolimus-induced cardiotoxicity. Tacrolimus produces ROS, which ultimately leads to oxidative stress inside the cell. High ROS generation can potentially harm cellular constituents and trigger NF-κB, a transcriptional regulator of inflammation. An inflammatory response is promoted by NF-κB activation, which stimulates the transcription of pro-inflammatory cytokines like IL-6 and TNF-α [48]. Besides this, tacrolimus may affect endothelial function by decreasing the synthesis of the anti-inflammatory and vasodilator molecule called NO. Endothelial dysfunction contributes to inflammation by increasing vascular permeability and encouraging inflammatory cells (TNF-α, IL-6) to adhere to the endothelium [49]. This allows the cells to migrate into cardiac tissue. Numerous studies suggested that TNF-α is the primary modulator of inflammatory responses after tacrolimus exposure, accounting for increasing levels of cytokines and chemokines. Dehghani et al. showed the effect of quercetin in a post-myocardial infarction patient. This study shows that quercetin has increased the total antioxidant capacity and downregulates the level of TNF-α in the quercetin-treated group as compared to the placebo control [50]. Our study found that rats given tacrolimus had considerably elevated levels of TNF-α and IL-6, indicating the presence of inflammatory cytokines that cause injury to cardiac cells. Fascinatingly, the levels of these inflammatory markers decrease after quercetin administration. This decrease in the levels of inflammatory cytokines indicates the cardioprotective potential of quercetin against tacrolimus-induced cardiotoxicity.

The histological architectural analysis shows visible changes in tacrolimus-treated animals like vacuole formation, inflammatory cell infiltration, and hypertrophy of cardiac tissue. The administration of a lower dose (100 mg/kg) of quercetin demonstrated few inflammatory cells with small vacuoles and cardiac hypertrophy. However, the administration of a higher dose of quercetin (200 mg/kg) remarkably counteracted the changes in the structural integrity of cardiac tissue, thus demonstrating cardioprotective potential. The result of this study significantly illustrates the protective potential of quercetin in tacrolimus-induced cardiotoxicity.

## 5 Conclusions

Our investigation concludes that quercetin has the potential to modulate the levels of cytokines and cardiac markers. The present study revealed the potential protective effect of quercetin in tacrolimus-induced cardiotoxicity due to its free radical scavenging and antioxidant properties. Quercetin restored the structural and functional changes of cardiac tissue damage caused due to tacrolimus administration. Therefore, it may be concluded that quercetin might be used in addition to other therapies to help control cardiotoxicity related to tacrolimus treatments during organ transplant therapy. Additional clinical research study is needed to investigate its clinical significance.
